# Comparison of choriocapillaris perfusion between swept-source optical coherence tomography angiography and spectral-domain optical coherence tomography angiography in five different choriocapillaris slabs in patients with intermediate age-related macular degeneration

**DOI:** 10.1007/s00417-025-06874-x

**Published:** 2025-06-14

**Authors:** Nuria Torrell-Belzach, Eric H. Souied, Hoang Mai Le, Manal Benlahbib, Zoe Dobbels, Ambroisine Erasti Gounfle, Xavier X. Wang, Camille Jung, Alexandra Miere

**Affiliations:** 1 University of Paris Est - Creteil, Creteil, France; 2 Clinical Research Center, CHIC, Creteil, France; 3 Department of Ophthalmology, CHI Créteil, 40 Avenue de Verdun, Creteil Cedex, 94000 France

**Keywords:** Choriocapillaris, Flow deficit, Intermediate age-related macular degeneration, Optical coherence tomography angiography

## Abstract

**Purpose:**

To analyze choriocapillaris (CC) perfusion using swept-source optical coherence tomography angiography (SS-OCTA) and spectral-domain optical coherence tomography angiography (SD-OCTA) in five CC slabs in patients with intermediate age-related macular degeneration (iAMD).

**Methods:**

This is a cross-sectional study of 23 eyes of 20 patients who underwent three 3 × 3 mm OCTA, one with SS-OCTA and two with SD-OCTA, a non-averaged scan (V1) and a four-scan volume (V4). Percentage of flow deficits (FD%), average size of FD (µm2), total area of FD (mm2) and number of FD were calculated in different CC slabs (automatic, 11–21 μm, 21–31 μm, 31–41 μm, 16–31 μm).

**Results:**

There was a statistically significant difference in all parameters and all slabs analyzed, when comparing SS-OCTA versus SD-OCTA V1 and SD-OCTA V1 versus SD-OCTA V4. Nevertheless, when comparing SS-OCTA versus SD-OCTA V4, significant differences were only found for the automatic and the 31–41 μm slab. When comparing the FD% between different slabs on the same device, significant differences were also found.

**Conclusion:**

Quantification of CC FD% is impacted by the CC slab, the type of OCTA used, and volume averaging in SD-OCTA. Given the significant impact on quantitative results, comparisons between studies and instruments and/or with/without averaging are difficult, even at the same slab depth.

## Key messages

*****What is known***:**

- Choriocapillaris (CC) flow impairment quantitative analysis by optical coherence tomography angiography (OCTA) is highly variable, depending on the type of device used, the selected choriocapillaris slab, the thresholding and the compensation method used.

*****What is new***:**

- To the best of our knowledge, this is the first study which analyzes four parameters of CC flow impairment on five different CC slabs with different OCTA devices to expand the knowledge of CC flow analysis.
- When comparing swept-source optical coherence tomography angiography (SS-OCTA) versus four-scan volume spectral-domain optical coherence tomography angiography (SD-OCTA V4), significant differences were only found for two out of five CC slabs for all four parameters analyzed.

## Introduction

The choriocapillaris (CC) is a single layer of fenestrated capillary network located inner in the choroid posterior to the Bruch’s membrane (BM), with a thickness of approximately 10–30 μm in healthy eyes [1]. Its functions consist of nutrition and metabolic exchange to the retinal pigment epithelium (RPE) and outer retina. Thus, damage of the CC is directly related to an imbalance of these structures, leading to an ischemic environment that is propitious to pathological changes in the retina [2]. CC flow decreases at the level of the fovea with aging, and this is more evident in patients with age-related macular degeneration (AMD) [3]. Although the pathogenic mechanism of AMD is not completely elucidated, the complex formed by photoreceptors, RPE, BM and CC are known to undergo progressive degeneration in AMD [4]. Hence, it is hypothesized that multiple risk factors could induce inflammation leading to RPE alteration and imbalance of the CC flow. Nevertheless, it is still a matter of debate whether AMD initiates in the CC or in the RPE.

One of the challenges of assessing the alterations of CC flow in both healthy subjects and AMD eyes has been limited by imaging techniques. In detail, the CC is not well visualized neither with fluorescein or indocyanine angiography [5], nor with optical coherence tomography (OCT). The advent of optical coherence tomography angiography (OCTA) has changed that, and this novel imaging technique has become essential in the analysis of the CC, as it provides a depth-resolved, high-resolution, and non-invasive in vivo visualization of this structure. Nevertheless, while a detailed qualitative analysis is possible with OCTA, quantification of the CC (non-)flow can be challenging due to motion artifacts, segmentation errors, masking effect, difference in CC slab, instrument, and signal quality [6]. Swept-source OCTA (SS-OCTA) is usually preferred to spectral-domain OCTA (SD-OCTA) for the analysis of deep retinal and choroidal structures, considering that the shorter wavelength of SD-OCTA is scattered by the RPE with less light penetration into the choroid.

In the last years, we have witnessed an increasing interest of the CC analysis with OCTA in retinal diseases, such as in AMD, diabetic retinopathy, pachychoroid spectrum disease, retinitis pigmentosa, ocular inflammation, hydroxychloroquine toxicity [7], Stargardt disease [8], and angioid streaks [9]. In the particular case of AMD, the OCTA analysis of the CC has demonstrated that CC dysfunction is a trigger factor for all forms of AMD or a coadjuvant factor for its progression [10]. In intermediate AMD (iAMD), the impairment of the CC flow has been identified in eyes that progress to a complete RPE and outer retinal atrophy (cRORA) [11] and, in advanced dry AMD [12]. Thus, there is an increasing interest in the potential role of CC flow impairment as a biomarker for progression and disease severity in AMD.

The primary aim of this study was to quantitatively determine CC flow impairment by measuring four different parameters, i.e.: the percentage of flow deficits (FD %), the average size of FD (µm2), the total area of FD (mm2), and the number of FD in 5 different CC slabs using both SS-OCTA (PLEX Elite 9000, Carl Zeiss Meditec Inc., Dublin, CA, USA) and SD-OCTA (Solix Fullrange, Optovue Inc, Freemont CA, USA) in patients with iAMD. For the SD-OCTA, two different image acquisition modes were performed, a non-averaged scan (V1) and a fast automated four scan volume (V4).

## Methods

This is a prospective, observational, cross-sectional study of consecutive patients with clinical diagnosis of iAMD in at least one eye, defined as at least one drusen greater than 125 μm with or without pigmentary abnormalities, in accordance with the Beckman classification [13]. They were examined at the Department of Ophthalmology at Creteil Hospital, a tertiary center in France, between April 2022 and January 2023.

All selected patients underwent best corrected visual acuity (BCVA) assessment, slit-lamp and fundus examination, an SD-OCT volume scan (Heidelberg Engineering, Heidelberg, Germany), and three different 3 × 3 mm OCTA acquisitions centered on the fovea were performed sequentially, with minimal time delay between them : one with SS-OCTA (PLEX Elite 9000, Carl Zeiss Meditec Inc., Dublin, CA, USA) and the other two with SD-OCTA (Solix Fullrange, Optovue Inc, Freemont CA, USA), including a V1 and V4 examination. Exclusion criteria included: previous treatment in the study eye with photodynamic therapy, presence of reticular pseudodrusen, OCTA acquisitions not well centered on the fovea, significant motion artifacts, any other retinal diseases other than AMD, glaucoma or optic neuropathy, refractive errors above 6 diopters, systemic illness treated with medication with potential retinal toxicity, poor quality scans (signal strength index < 8), differences in signal strength > 1 between the three acquisition modes, media opacities interfering with retinal imaging.

### OCTA image processing

All patients underwent a field of view of 3 × 3 mm scan centered on the fovea (400 pixels x400 pixels for the SD-OCTA and 1024 pixels x1024 pixels for the SS-OCTA). These images were assessed after removal of retinal projection artifacts using the built-in software in both OCTA devices. The SD-OCTA instrument Solix Fullrange (Optovue Inc, Freemont CA, USA) uses a laser light source with a central wavelength of 840 nm, a lateral resolution of 15 μm, an axial resolution of 5 μm and an acquisition rate of 120,000 A-scans per second. The SS-OCTA instrument PLEX Elite 9000 (Carl Zeiss Meditec Inc., Dublin, CA, USA) has a swept laser light with a central wavelength of 1050 nm, a lateral resolution of 20 μm, an optical axial resolution of 6.3 μm and acquisition rate of 100,000 A-scans per second. FastTrac motion software was used for the acquisition of all images acquired by SS-OCTA to mitigate motion artifacts. When present, segmentation errors were corrected manually.

The CC *en face* structural and flow images from each one of the five different CC slabs (automatic, 11–21 μm underneath the BM, 21–31 μm underneath the BM, 31–41 μm underneath the BM and 16–31 μm underneath the BM) were extracted from both OCTA devices and imported into FIJI Software (National Institute of Health, Bethesda, Maryland, USA). The automatic slabs were defined differently on the SD-OCTA and SS-OCTA devices, as follows: the automatic CC slab was defined as 9–31 μm underneath the BM for the SD-OCTA, and as 0–20 μm underneath the BM for the SS-OCTA device. An algorithm was used to compensate for the signal attenuation from changes in the RPE/BM/CC complex. Hence, as previously reported, the Fiji “invert” function to the OCT CC structural *en face* slab was used. Then, a Gaussian blur filter was applied for image smoothing followed by a multiplication between the processed CC structural *en face* image and the CC *en face* flow image to obtain a compensated CC *en face* flow image with the “Image Calculator” function. Finally, the Phansalkar local thresholding binarization method with a window radius of 4 pixels was applied to the compensated CC *en face* flow image, which converts grey scale into a black and white pixel image.

Consecutively, the “Analyze Particles” function enables the quantification of parameters to represent CC flow impairment: the percentage, the number, the size, and the total area of the FD. The CC FD% is defined as the percentage of pixels representing flow deficits relative to the total quantifiable area in the chosen region centered on the fovea. The number of FDs (FDN) is the number of contiguous black pixels, which represent the FD. The mean size of the FDs (MFDS) is the mean in square micrometers (µm^2^) of the contiguous black pixel area.

**Fig. 1:**
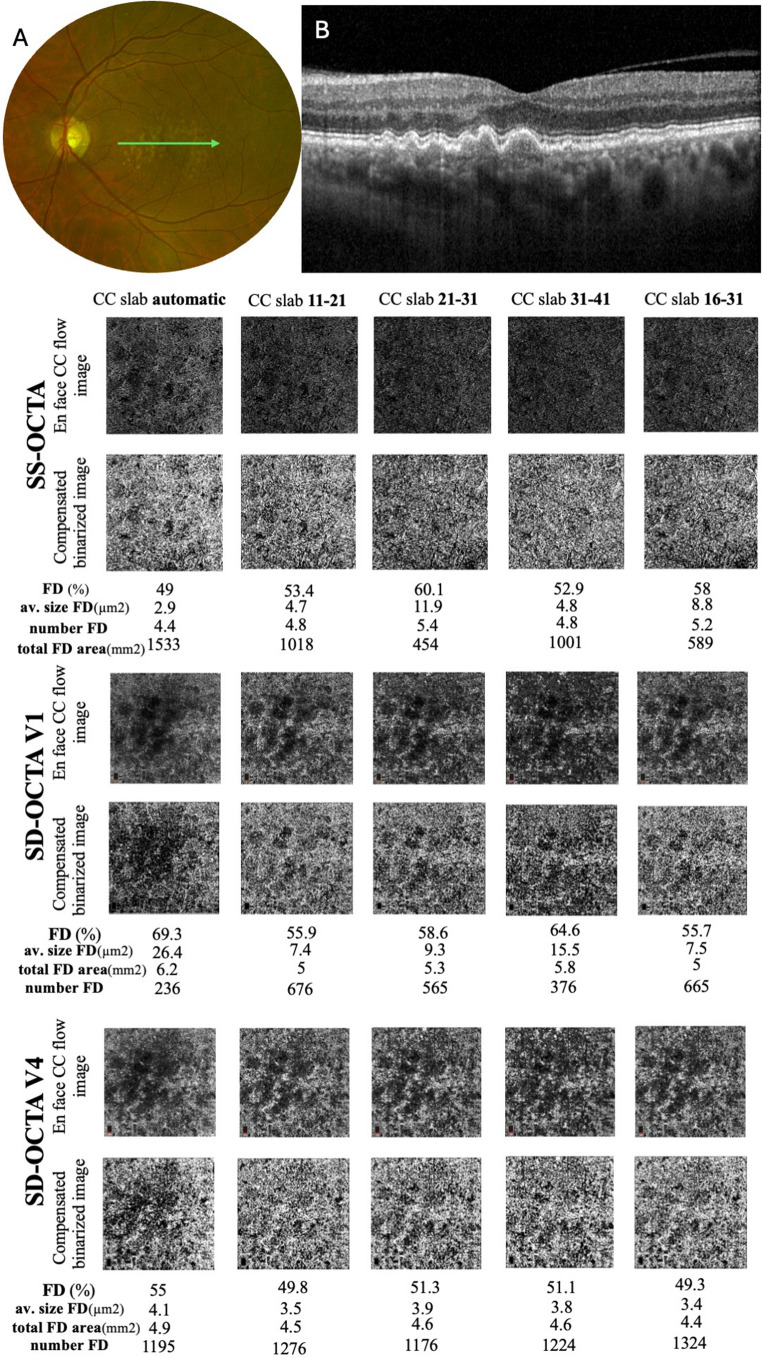
Multimodal imaging and choriocapillaris (CC) analysis of the left eye of a 74 year-old male subject with intermediate age-related macular degeneration (iAMD). Fundus photography shows macular soft drusen (**A**) and macular spectral-domain optical coherence tomography (SD-OCT) scan centered on the fovea (**B**), demonstrates soft drusen and small drusenoid retinal pigment epithelium detachments. Lower panels show the comparison of different CC parameters at the level of different CC slabs in this patient. FD: flow deficits; SS-OCTA: swept-source optical coherence tomography angiography; SD-OCTA V1: non-averaged spectral-domain optical coherence tomography angiography scan; SD-OCTA V4: averaged spectral-domain optical coherence tomography angiography using four scan volumes. Av. size FD: average size of FD

The total area of FD represents the area of all the FDs, given in square millimeters (mm^2^). All these four parameters were calculated in the five different CC slabs previously described (Fig. 1).

### Statistical analysis

Continuous data is expressed as mean ± standard deviation. The intraclass correlation coefficient (ICC2) was used to measure the degree of agreement between devices and to assess the reliability of measurements taken by each device. A mixed linear model with a random factor was performed to evaluate the significance of the device and slab factors. Post-hoc analyses using Tukey’s test for ANOVA were conducted to determine which pairs of devices and slabs groups differ significantly. A *P* value < 0.05 was considered to be statistically significant.

## Results

**Fig. 2:**
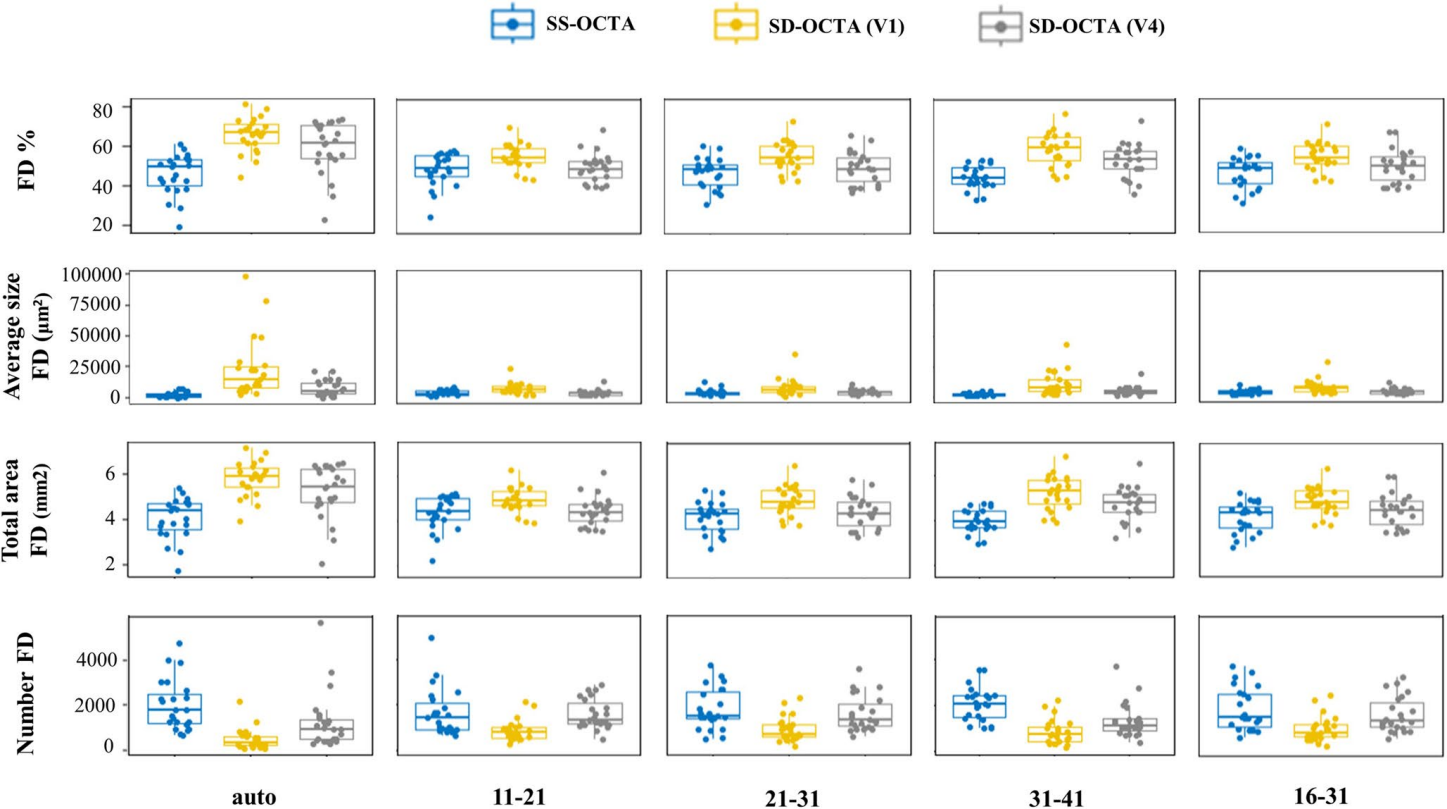
Graphical distribution of the four quantitative variables of choriocapillaris (CC) analysis: upper row percentage of FD (FD%), second row FD average size in µm2, third row total area of FD in mm2 and bottom row number of FD, computed on different slabs (each column represents one of the five chosen CC slabs) and on the different devices (SS-OCTA, SD-OCTA V1 and SD-OCTA V4). SS-OCTA: swept-source optical coherence tomography angiography; SD-OCTA V1: non-averaged spectral-domain optical coherence tomography angiography scan; SD-OCTA V4: fast automated spectral-domain optical coherence tomography angiography four scan volumes

Twenty-three eyes of twenty patients were included (mean age of 76.8 +/- 6.2 years, 6 males and 14 females). There was no statistically significant difference between the signal strength on each device (*P* = 0.09) (Fig. 2).

### Comparison between devices

#### Comparison for each variable on each slab between SS-OCTA and SD-OCTA V1

There was a statistically significant difference in terms of CC FD%, average size of FD, total area of FD and number of FD for all five CC slabs, when comparing SS-OCTA versus SD-OCTA V1 (*P* < 0.05, Wilcoxon signed-rank test) (Table 1).

**Table 1:** Quantitative data of the percentage of flow deficits, the average size of flow deficits in µm2, the total area of flow deficits in mm2, and the number of flow deficits for each of the five different choriocapillaris selected slabs

Parameter	CC slab	SS-OCTA	SD-OCTA V1	SD-OCTA V4	SS-OCTA vs. SD-OCTA V1 (*p* value)	SS-OCTAvs. SD-OCTA V4 (*p* value)	SD-OCTA V1 vs. SD-OCTA V4 (*p* value)
Percentage FD (FD%)	Auto	45.38 ± 10.08	65.10 ± 8.51	58.55 ± 13.10	**< 0.05**	**< 0.05**	**< 0.05**
	11–21	48.25 ± 8.39	54.66 ± 6.22	49.14 ± 7.21	**< 0.05**	0.71	**< 0.05**
	21–31	46.56 ± 7.52	54.98 ± 7.31	49.02 ± 7.95	**< 0.05**	0.56	**< 0.05**
	31–41	44.17 ± 5.76	58.01 ± 8.33	52.41 ± 8.39	**< 0.05**	**< 0.05**	**< 0.05**
	16–31	46.30 ± 7.50	54.32 ± 6.99	49.50 ± 8.29	**< 0.05**	0.30	**< 0.05**
Average size of FD (µm2)	Auto	3018.24 ± 2099.78	24148.48 ± 24287.23	8337.45 ± 6490.23	**< 0.05**	**< 0.05**	**< 0.05**
	11–21	3682.10 ± 2110.82	7401.20 ± 4626.30	3554.61 ± 2535.53	**< 0.05**	0.27	**< 0.05**
	21–31	3374.54 ± 2734.85	7431.20 ± 6986.12	3600.88 ± 2200.15	**< 0**,**05**	0.69	**< 0.05**
	31–41	2348.40 ± 1249.21	10977.93 ± 9794.81	5023.95 ± 3729.30	**< 0.05**	**< 0.05**	**< 0.05**
	16–31	3096.45 ± 2100.21	7346.80 ± 5775.65	3774.75 ± 2464.76	**< 0.05**	0.34	**< 0.05**
Total area of FD (mm2)	Auto	4.08 ± 0.91	5.86 ± 0.77	5.27 ± 1.18	**< 0.05**	**< 0.05**	**< 0.05**
	11–21	4.34 ± 0.76	4.92 ± 0.56	4.42 ± 0.65	**< 0.05**	0.70	**< 0.05**
	21–31	4.19 ± 0.67	4.95 ± 0.66	4.41 ± 0.72	**< 0.05**	0.56	**< 0.05**
	31–41	3.98 ± 0.52	5.22 ± 0.75	4.72 ± 0.76	**< 0.05**	**< 0.05**	**< 0.05**
	16–31	4.17 ± 0.68	4.89 ± 0.63	4.46 ± 0.75	**< 0.05**	0.30	**< 0.05**
Number of FD	Auto	1987.09 ± 1114.07	501.96 ± 455.74	1256.65 ± 1592.26	**< 0.05**	**< 0.05**	**< 0.05**
	11–21	1661.30 ± 1036.97	876.57 ± 464.40	1592.26 ± 657.53	**< 0.05**	0.71	**< 0.05**
	21–31	1799.26 ± 897.19	926.57 ± 540.35	1601.65 ± 772.07	**< 0.05**	0.58	**< 0.05**
	31–41	2041.65 ± 740.42	824.83 ± 554.65	1313.87 ± 754.59	**< 0.05**	**< 0.05**	**< 0.05**
	16–31	1836.65 ± 941.40	971.52 ± 565.80	1583.26 ± 780.10	**< 0.05**	0.43	**< 0.05**

*CC* choriocapillaris, *FD* flow deficit, *SS-OCTA* swept-source optical coherence tomography angiography, *SD-OCTA V1* non-averaged spectral-domain optical coherence tomography angiography scan, *SD-OCTA V4* fast automated spectral-domain optical coherence tomography angiography four scan volumes

#### Comparison for each variable on each slab between SS-OCTA and SD-OCTA V4

A statistical significance was found only for the automatic slab and the 31–41 μm slab when comparing SS-OCTA versus SD-OCTA V4 for all four parameters analyzed (*P* < 0.05, Wilcoxon signed-rank test) (Table 1).

#### Comparison for each variable on each slab between SD-OCTA V1 and SD-OCTAV4

There was also a statistically significant difference in all four parameters analyzed for all the five CC slabs, when comparing SD-OCTA V1 versus SD-OCTA V4 (*P* < 0.05, Wilcoxon signed-rank test) (Table 1).

### Comparison of the percentage of flow deficits between different slabs on the same device

#### SS-OCTA

There was a statistically significant difference in terms of CC FD% between the CC slab 11–21 μm versus 31–41 μm, when using the SS-OCTA device (Table 2).

**Table 2:** Comparison of the percentage of flow deficits between the five different choriocapillaris slabs on the same device

	CC slab	Auto	11–21	21–31	31–41	16–31
SS-OCTA FD%	Auto	*N*/A	0.12	0.52	0.51	0.61
	11–21	0.12	N/A	0.36	**< 0.05**	0.29
	21–31	0.52	0.36	N/A	0.19	0.89
	31–41	0.51	**< 0.05**	0.19	N/A	0.25
	16–31	0.61	0.29	0.89	0.25	N/A
SD-OCTA V1 FD%	Auto	N/A	**< 0.05**	**< 0.05**	**< 0.05**	**< 0.05**
	11–21	**< 0.05**	N/A	0.86	0.07	0.85
	21–31	**< 0.05**	0.86	N/A	0.10	0.72
	31–41	**< 0.05**	0.07	0.10	N/A	**< 0.05**
	16–31	**< 0.05**	0.85	0.72	**< 0.05**	N/A
SD-OCTA V4 FD%	Auto	N/A	**< 0.05**	**< 0.05**	**< 0.05**	**< 0.05**
	11–21	**< 0.05**	N/A	0.95	0.08	0.84
	21–31	**< 0.05**	0.95	N/A	0.07	0.79
	31–41	**< 0.05**	0.08	0.07	N/A	0.11
	16–31	**< 0.05**	0.84	0.79	0.11	N/A

*CC* Choriocapillaris, *FD%* percentage of flow deficit, *SS-OCTA* swept-source optical coherence tomography angiography, *SD-OCTA V1* non-averaged spectral-domain optical coherence tomography angiography scan, *SD-OCTA V4* fast automated spectral-domain optical coherence tomography angiography four scan volumes. *N*/*A* Not applicable

#### SD-OCTA V1

There was a statistically significant difference in terms of CC FD% between the CC automatic slab and all the other four slabs and between the slab 31–41 μm versus 16–31 μm, when using the acquisition mode SD-OCTA V1 (Table 2).

#### SD-OCTA V4

There was a statistically significant difference in terms of CC FD% between the CC automatic slab and all the other four slabs, when using the acquisition mode SD-OCTA V4 (Table 2).

Moreover, the concordance analysis (ICC2) for the FD% between the different slabs on the SS-OCTA was: 0.93 (95% confidence interval: 0.86–0.96) for the SD-OCTA V1 was 0.90 (95% confidence interval: 0.66–0.96) and for the SD-OCTA V4 was 0.91 (95% confidence interval: 0.78–0.96).

## Discussion

In this study, we showed that in iAMD eyes there were significant differences in terms of CC flow impairment between different OCTA devices with different acquisition modes (SS-OCTA versus SD-OCTA V1 and V4) and between different CC slab depths on the same device. To the best of our knowledge, this is the first study to quantitatively compare CC analysis on different slabs and different devices, thus expanding the knowledge of the role (and limitations) of OCTA in CC analysis in iAMD.

A clear trend of variability in the quantitative measurements of choriocapillaris flow deficits (CC FD) across the three OCTA modalities has been observed, particularly within the automatically generated CC slab (Fig. 2). Notably, SS-OCTA consistently yields lower FD% values compared to SD-OCTA V1 and V4. SD-OCTA V1 tends to overestimate both FD% and total FD area, especially within the automatic CC slab, likely due to its shorter wavelength and increased susceptibility to artifacts. SD-OCTA V4 produces intermediate measurements but generally aligns more closely with V1 in most slab comparisons. These findings indicate a systematic variation in FD% quantification based on the OCTA modality employed.

OCTA is still a very new technology, and inappropriate measures could lead to inappropriate analysis of the CC, due to our limited understanding, myriads of variables to be considered, and intrinsic limitations of current OCTA devices. Thus, CC flow impairment quantitative analysis by OCTA is highly variable, depending not only on the type of device used (SD-OCTA versus SS-OCTA), but also on the selected CC slab, the thresholding and the compensation method used. For instance, Byon et al. [14] demonstrated in 2019 that shifts as small as 4 μm regarding the CC slab position could lead to significant differences in CC FDs measurements in healthy eyes with SS-OCTA. This study also demonstrated that slabs near the BM were more propense to be confounded by segmentation artifacts, due to inadvertent inclusion of the RPE layer [14]. Current OCT devices cannot distinguish RPE (11–14 μm thick) from the BM (2–6 μm thick), unless they are pathologically separated. Therefore, if the RPE (upper boundary of the RPE/BM complex) is being segmented, the upper boundary of the CC slab should be placed in theory 16 μm beneath the segmentation line. If the BM (lower boundary of the RPE/BM complex) is being segmented, the upper boundary of the CC should be placed 4 μm below the segmentation line.

Nevertheless, many different CC slabs have been used in several studies aiming to assess CC flow impairment either in healthy or diseased eyes. In 2021, Chu et al. [15] published the first evidence-based guideline for imaging the CC using SS-OCTA, where the authors emphasized that the most important step to analyze CC flow is to provide accurate segmentation of the CC. Following their recommendations, recent studies have chosen the 16 μm thick CC slab, from 4 μm to 20 μm beneath the BM [16–19], using an SS-OCTA instrument. Zhang et al. [2] et Rinella et al. [12] used the slab from the outer boundary of BM to 20 μm beneath BM, while Cheng et al. [5] defined the boundaries of the CC from the BM to 10.4 μm below the BM. Furthermore, Le et al. [9] used a 15 μm thick slab, starting 16 μm under the RPE/BM, as it was described by Chu et al. [20] in 2019, while other studies chose a 10 μm thick CC slab starting 31 μm posterior to the RPE-fit reference, which is really an inner choroidal slab presumed to capture the projection artifact of the CC [21–23]. More recently, Alagorie et al. [24] used three different CC slab selection in a study published in 2023: 11–21 μm, 21–31 μm and 31–41 μm below the RPE band centerline to demonstrate the differences between different segmentations of the CC slab. As this multitude of definitions of CC slab found in recent literature show, there is still no consensus in terms of choice of the appropriate CC segmentation, and therefore comparisons between studies with different CC slabs (and different image processing algorithms) are really challenging, if not impossible.

We assumed in our study that the thickness of the CC slab should consider that the axial resolution of OCT is approximately 6 μm, so a CC slab of 10–20 μm thick beneath the BM should provide the most accurate CC *en face* flow OCTA image. Thus, our more superficial CC slab chosen in the current study is 11–21 μm from the BM, which takes also into consideration that a superficial slab presumed to be the anatomical position of the CC is more susceptible to having segmentation errors, which may lead to an inclusion of the RPE-BM complex, as Byon et al. [14] reported.

Another important parameter to accurately analyze the CC is the ICD, which has been defined as the averaged distance from one center of a capillary lumen to another. Thus, the ICD would be the sum of the width of one capillary and the width of a physiological flow void [15]. The diameter of a single CC vessel in the macular region has been demonstrated to average 16–20 μm with an edge-to-edge vessel distance of 5–20 μm [25]. Therefore, the ICD is about 21–40 μm, considering one vessel with plus one flow void. Thus, choosing a radius pixel of 4 excludes all FD < 27 μm in the analysis that can be within the range of normal ICD [26]. Hence, the window radius parameter used in the Phansalkar method should be considered in CC assessment. As previously reported by Chu et al. [15], a radius of 4–8 pixels should be chosen for a 3 × 3 mm scan, and a radius of 2–4 pixels for a 6 × 6 mm scan, considering the ICD. Moreover, different studies have shown that the size of the radius affects the repeatability and analysis of the CC FD% which are based on the Phansalkar method, with a higher CC FD% as the radius increases [26]. Nevertheless, there is no consensus yet of the best radius for this method.

It is also important to highlight that there are other CC binarization techniques different from Phansalkar local thresholding method: the global thresholding method and the fuzzy C -means algorithm. The discrepancy between different binarization techniques between studies makes comparison between studies impossible as well [15]. Nevertheless, as Laiginhas et al. [27] stated in 2020, local thresholding methods are superior to the global ones for quantifying the CC and they should be preferred.

Given the above, our study has numerous limitations. On one hand, the relatively small sample size influenced by the strict exclusion criteria, the inclusion of both eyes of the same patient in some cases, and the fact that a test of repeatability of the measures was not performed. Moreover, the CC flow impairment was not analyzed in different regions relative to the fovea or to the drusen disposition. However, we believe that the present study has important strengths, such as its prospective design, the high signal strength of the OCTA images (> 7/10), and the analysis of 5 different slabs at the level of the CC with both SS-OCTA and SD-OCTA devices.

In conclusion, our study demonstrates the importance of selecting a specific CC slab to compare measures between studies, as the quantitative analyses of the CC perfusion parameters are significantly impacted by the selected CC slab. Moreover, not only the type of OCTA instrument used (SS-OCTA vs. SD-OCTA), but also different volume averaging in SD-OCTA (V1 and V4) comparisons show statistically significant differences.

There are of course inherent limitations of cross-device comparisons in this study. Differences in imaging technology, acquisition protocols, and segmentation algorithms across OCTA modalities introduce variability that may confound direct comparisons. Thus, our results should not be interpreted as definitive conclusions regarding the superiority or accuracy of any specific device. Instead, they intend to serve as foundational data to inform and guide future research of choriocapillaris flow deficit quantification.

Therefore, in studies involving the CC, it is crucial to describe the best binarization thresholding method and the appropriate CC slab for each OCTA instrument to better compare results between studies, as there is no consensus between experts yet. For research purposes, it is important to use the same binarization thresholding method, the same device, and the same CC slab to acquire reliable results for the longitudinal follow-up of the patients. In addition, larger longitudinal studies are needed to better understand the role of CC perfusion in iAMD and advanced AMD to help redefine AMD pathogenesis, and consequently find new prognostic biomarkers, which could lead to new therapeutic approaches to delay or prevent AMD progression.

## References

[1] Singh RB, Perepelkina T, Testi I et al (2022) Imaging-based assessment of choriocapillaris: a comprehensive review. Semin Ophthalmol 38(5):405–426. 10.1080/08820538.2022.2109939 35982638 10.1080/08820538.2022.2109939

[2] Zhang Q, Zheng F, Motulsky EH et al (2018) A novel strategy for quantifying choriocapillaris flow voids using swept-source OCT angiography. Invest Ophthalmol Vis Sci 59(1):203–211. 10.1167/iovs.17-22953 29340648 10.1167/iovs.17-22953 PMC5770182

[3] Lutty G, Grunwald J, Majji AB et al (1999) Changes in choriocapillaris and retinal pigment epithelium in age-related macular degeneration. Mol Vis 3:5:35 10562659

[4] Bhutto I, Lutty G (2012) Understanding age-related macular degeneration (AMD): relationships between the photoreceptor/retinal pigment epithelium/bruch’s membrane/choriocapillaris complex. Mol Aspects Med 33(4):295–317. 10.1016/j.mam.2012.04.005 22542780 10.1016/j.mam.2012.04.005 PMC3392421

[5] Cheng W, Song Y, Lin F et al (2022) Choriocapillaris flow deficits in normal Chinese imaged by swept-source optical coherence tomographic angiography. Am J Ophthalmol 235:143–153. 10.1016/j.ajo.2021.09.018 34582767 10.1016/j.ajo.2021.09.018

[6] Spaide RF, Fujimoto JG, Waheed NK (2015) Image artifacts in optical coherence tomography angiography. Retina 35(11):2163–2180. 10.1097/IAE.0000000000000765 26428607 10.1097/IAE.0000000000000765 PMC4712934

[7] Halouani S, Le HM, Cohen SY et al (2022) Choriocapillaris flow deficits quantification in hydroxychloroquine retinopathy using swept-source optical coherence tomography angiography. J Pers Med 12(9):1445. 10.3390/jpm12091445 36143230 10.3390/jpm12091445 PMC9503306

[8] Mastropasqua R, Senatore A, Di Antonio L et al (2019) Correlation between choriocapillaris density and retinal sensitivity in Stargardt disease. J Clin Med 8(9):1432. 10.3390/jcm8091432 31510083 10.3390/jcm8091432 PMC6780313

[9] Le HM, Souied EH, Halouani S et al (2022) Quantitative analysis of choriocapillaris using swept-source optical coherence tomography angiography in eyes with angioid streaks. J Clin Med 11(8):2134. 10.3390/jcm11082134 35456229 10.3390/jcm11082134 PMC9026537

[10] Flores R, Carneiro Â, Neri G et al (2022) Choroidal vascular impairment in intermediate age-related macular degeneration. Diagnostics 12(5):1290. 10.3390/diagnostics12051290 35626445 10.3390/diagnostics12051290 PMC9141612

[11] Corvi F, Corradetti G, Tiosano L et al (2021) Topography of choriocapillaris flow deficit predicts development of neovascularization or atrophy in age-related macular degeneration. Graefe’s Arch Clin Exp Ophthalmol 259(10):2887–2895. 10.1007/s00417-021-05167-3 33900443 10.1007/s00417-021-05167-3

[12] Rinella NT, Zhou H, Wong J et al (2022) Correlation between localized choriocapillaris perfusion and macular function in eyes with geographic atrophy. Am J Ophthalmol 234:174–182. 10.1016/j.ajo.2021.08.007 34437870 10.1016/j.ajo.2021.08.007

[13] Ferris FL, Wilkinson CP, Bird A et al (2013) Clinical classification of age-related macular degeneration. Ophthalmology 120(4):844–851. 10.1016/j.ophtha.2012.10.036 23332590 10.1016/j.ophtha.2012.10.036 PMC11551519

[14] Byon I, Nassisi M, Borrelli E, Sadda SR (2019) Impact of slab selection on quantification of choriocapillaris flow deficits by optical coherence tomography angiography. Am J Ophthalmol 208:397–405. 10.1016/j.ajo.2019.08.026 31493401 10.1016/j.ajo.2019.08.026

[15] Chu Z, Zhang Q, Gregori G et al (2021) Guidelines for imaging the choriocapillaris using OCT angiography. Am J Ophthalmol 222:92–101. 10.1016/j.ajo.2020.08.045 32891694 10.1016/j.ajo.2020.08.045 PMC7930158

[16] Li J, Liu Z, Lu J et al (2023) Decreased macular choriocapillaris perfusion in eyes with macular reticular pseudodrusen imaged with swept-source OCT angiography. Invest Ophthalmol Vis Sci 64(4):15. 10.1167/iovs.64.4.15 37052925 10.1167/iovs.64.4.15 PMC10103727

[17] Cabral D, Fradinho AC, Zhang Y et al (2023) Quantitative assessment of choriocapillaris flow deficits and type 1 macular neovascularization growth in age – related macular degeneration. Sci Rep 13:1–8. 10.1038/s41598-023-35080-0 37236984 10.1038/s41598-023-35080-0 PMC10220043

[18] Wu Z, Zhou X, Chu Z et al (2022) Impact of reticular pseudodrusen on choriocapillaris flow deficits and choroidal structure on optical coherence tomography angiography. Investig Ophthalmol Vis Sci 63(12):1. 10.1167/iovs.63.12.1 10.1167/iovs.63.12.1 PMC9639673 36318196

[19] Corvi F, Cozzi M, Corradetti G et al (2021) Quantitative assessment of choriocapillaris flow deficits in eyes with macular neovascularization. Graefe’s Arch Clin Exp Ophthalmol 259(7):1811–1819. 10.1007/s00417-020-05056-1 33417089 10.1007/s00417-020-05056-1

[20] Chu Z, Gregori G, Rosenfeld PJ, Wang RK (2019) Quantification of choriocapillaris with optical coherence tomography angiography: a comparison study. Am J Ophthalmol 208:111–123. 10.1016/j.ajo.2019.07.003 31323202 10.1016/j.ajo.2019.07.003 PMC6889046

[21] Byon I, Ji Y, Alagorie AR et al (2021) Topographic assessment of choriocapillaris flow deficits in the intermediate age-related macular degeneration eyes with hyporeflective cores inside drusen. Retina 41(2):393–401. 10.1097/IAE.0000000000002906 33475272 10.1097/IAE.0000000000002906

[22] Alagorie AR, Verma A, Nassisi M, Sadda SR (2019) Quantitative assessment of choriocapillaris flow deficits in eyes with advanced age-related macular degeneration versus healthy eyes. Am J Ophthalmol 205:132–139. 10.1016/j.ajo.2019.04.037 31078531 10.1016/j.ajo.2019.04.037

[23] Tiosano L, Corradetti G, Sadda SR (2021) Progression of choriocapillaris flow deficits in clinically stable intermediate age-related macular degeneration. Eye 35(11):2991–2998. 10.1038/s41433-020-01298-9 33414537 10.1038/s41433-020-01298-9 PMC8526707

[24] Alagorie AR, Corradetti G, Byon I et al (2024) Impact of slab selection on the relationship between choriocapillaris flow deficits and enlargement rate of geographic atrophy. Eye 38(5):847–852. 10.1038/s41433-023-02788-2 37865725 10.1038/s41433-023-02788-2 PMC10966059

[25] Olver JM (1990) Functional anatomy of the choroidal circulation: methyl methacrylate casting of human choroid. Eye 4(2):262–272. 10.1038/eye.1990.38 2379644 10.1038/eye.1990.38

[26] Chu Z, Cheng Y, Zhang Q et al (2020) Quantification of choriocapillaris with Phansalkar local thresholding: pitfalls to avoid. Am J Ophthalmol 213:161–176. 10.1016/j.ajo.2020.02.003 32059979 10.1016/j.ajo.2020.02.003 PMC7214221

[27] Laiginhas R, Cabral D, Falcão M (2020) Evaluation of the different thresholding strategies for quantifying choriocapillaris using optical coherence tomography angiography. Quant Imaging Med Surg 10(10):1994–2005. 10.21037/qims-20-340 33014731 10.21037/qims-20-340 PMC7495317

